# Multidisciplinary first-day consultation accelerates diagnostic procedures and throughput times of patients in a head-and-neck cancer care pathway, a mixed method study

**DOI:** 10.1186/s12913-018-3637-1

**Published:** 2018-10-29

**Authors:** Lidia S. van Huizen, Pieter U. Dijkstra, Bernard F. A. M. van der Laan, Harry Reintsema, Kees T. B. Ahaus, Hendrik P. Bijl, Jan L. N. Roodenburg

**Affiliations:** 1 Department of Oral and Maxillofacial Surgery, University of Groningen, University Medical Center Groningen, Groningen, The Netherlands; 2 Department of Quality and Patient Safety, University of Groningen, University Medical Center Groningen, Groningen, The Netherlands; 3 Center for Rehabilitation, University of Groningen, University Medical Center Groningen, Groningen, The Netherlands; 4 Department of Ear, Nose & Throat, University of Groningen, University Medical Center Groningen, Groningen, The Netherlands; 5 Faculty of Economics and Business, Centre of Expertise Healthwise, University of Groningen, University Medical Center Groningen, Groningen, The Netherlands; 6 Department of Radiotherapy, University of Groningen, University Medical Center Groningen, Groningen, The Netherlands

**Keywords:** First-day consultation, Oncology, Management care pathways, Critical pathways (MeSH), Process indicators, Mixed method study, Head and neck cancer

## Abstract

**Background:**

Head and neck cancers are fast growing tumours that are complex to diagnose and treat. Multidisciplinary input into organization and logistics is critical to start treatment without delay. A multidisciplinary first-day consultation (MFDC) was introduced to reduce throughput times for patients suffering from head and neck cancer in the care pathway. In this mixed method study we evaluated the effects of introducing the MFDC on throughput times, number of patient hospital visits and compliance to the Dutch standard to start treatment within 30 calendar-days.

**Methods:**

Data regarding ‘days needed for referral’, ‘days needed for diagnostic procedures’, ‘days to start first treatment’, and ‘number of hospital visits’ (process indicators) were retrieved from the medical records and analysed before and after implementation of the MFDC (before implementation: 2007 (*n* = 21), and after 2008 (*n* = 20), 2010 (*n* = 24) and 2013 (*n* = 24)). We used semi-structured interviews with medical specialists to explore a sample of outliers.

**Results:**

Comparing 2007 and 2008 data (before and after MFDC implementation), days needed for diagnostic procedures and to start first treatment reduced with 8 days, the number of hospital visits reduced with 1.5 visit on average. The percentage of new patients treated within the Dutch standard of 30 calendar-days after intake increased from 52 to 83%.

The reduction in days needed for diagnostic procedures was sustainable. Days needed to start treatment increased in 2013. Semi-structured interviews revealed that this delay could be attributed to new treatment modalities, patients needed more time to carefully consider their treatment options or professionals needed extra preparation time for organisation of more complex treatment due to early communication on diagnostic procedures to be performed.

**Conclusions:**

A MFDC is efficient and benefits patients. We showed that the MFDC implementation in the care pathway had a positive effect on efficiency in the care pathway. As a consequence, the extra efforts of four specialist disciplines, a nurse practitioner, and a coordinating nurse seeing the patient together during intake, were justified. Start treatment times increased as a result of new treatment modalities that needed more time for preparation.

## Background

The tumours in the head or neck region (nasal cavity, sinuses, lips, mouth, salivary glands, throat, or larynx) are fast growing tumours. This means that a long interval between the moment of referral and the start of the primary treatment (surgery, radiotherapy and/or chemotherapy) can lead to upstaging of the tumour with less chance on cure [1]. Because of the complexity of the diagnostic procedures and therapeutic modalities, head and neck cancer care is centralized in special multidisciplinary head and neck cancer centres [2]. Although the patient’s prognosis is determined by tumour stage, throughput time, defined as ‘day from first visit to day of start of treatment’ should be kept as short as possible [3, 4]. According to the Dutch Cooperative Head & Neck Group [5] treatment should start within 30 calendar-days after intake for 80% of new patients.

Until September 2007, the intake of head and neck patients at the University Medical Center Groningen (UMCG) was performed by the Department of Oral and Maxillofacial Surgery (OMS) and the Department of Ear, Nose & Throat (ENT), the front offices for the multidisciplinary head and neck centre. On the day of intake, the specialists or the nurse practitioner of the gate departments planned the diagnostic procedures, and two weeks after that, the diagnosis and treatment plan were discussed in the multidisciplinary meeting. In the meantime the involved supportive paramedical specialists, such as the dental team (special care dentist, oral hygienist), speech therapists, dieticians, and medical social workers, were consulted prior to the multidisciplinary meeting. This meeting was the first opportunity for a multidisciplinary discussion in the care pathway about treatment, based on written history, physical examination, laboratory data, and imaging. The patient was not present during the meeting.

In the Netherlands, the number of head and neck cancer cases increased between 1989 and 2016 [6] from 1934 to 2995 cases which is an increase of 55%. The highest increase is seen for patients over 75 years with 88% followed by the age group 60–74 years with 80% (Table 1). This increase and limited resources were reasons to improve the efficiency of diagnostics and treatment for patients and because the performance of the centre on throughput time was poor in 2007 (only 52% of patients started their treatment within the 30-day standard), the centre decided to implement a multidisciplinary first-day consultation (MFDC) to reduce the time to start treatment.

**Table 1:** Incidence rates Head & Neck Cancer in the Netherlands

Period	Number of cases per age category						Total	Dutch population
	0–14	15–29	30–44	45–59	60–74	75+		
1989	3	17	139	546	852	377	1934	14,805,240
1990	1	17	144	606	900	409	2077	14,892,574
2007	2	20	132	804	1109	587	2654	16,357,992
2008	5	26	133	849	1291	575	2879	16,405,399
2010	2	17	120	814	1326	596	2875	16,574,989
2013	0	25	91	776	1421	644	2957	16,779,575
2016	0	13	72	669	1531	710	2995	16,979,120

Whilst care pathways are organized with multidisciplinary patient meetings (MPM), but as evidence based guidelines are accepted to organize care, the added value of each MPM still should be proven [7]. Brunner et al. support this view in 2015 by explaining that the last 30 years multidisciplinary team meetings have become an essential component of tertiary-level decision-making in the treatment of malignancy [8]. It seems self-evident that the variety of specialist team members with their combined knowledge and expertise improve decision making and therefore a MPM is often described in guidelines as a structure indicator.

**Fig. 1:**
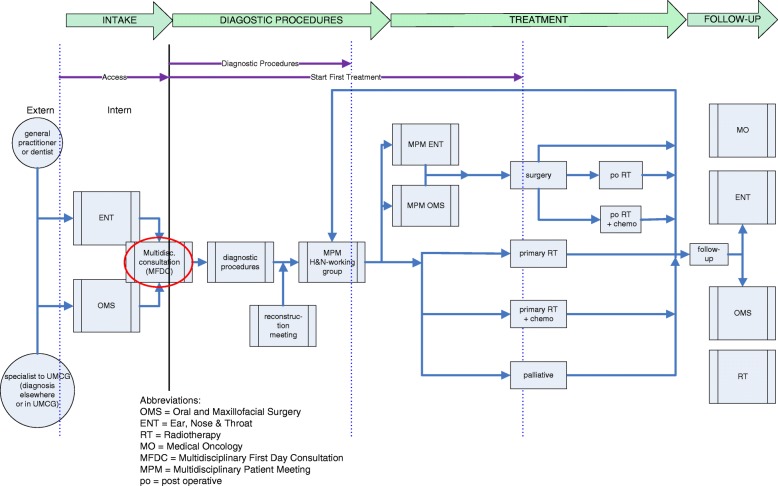
Care Pathway Head & Neck Oncology and throughput time definition. Legend Fig. 1. The care pathway consists of diverse personnel of four core departments (ENT, OMS, MO, and RT). The care pathway sub-processes are called ‘intake - diagnostic procedures – treatment – follow-up’. There are four treatment modalities: surgery, primary radiation, chemo and chemo-radiation. In the red circle the intervention: the MFDC

The research question is: what are the effects of the MFDC implementation on efficiency of the care pathway, measured as process indicators throughput times (referral, diagnostic procedures and start treatment) and number of hospital visits (Fig. 1).

## Methods

The MFDC was introduced in 2007 in the head and neck cancer care pathway using an ‘8-step method’ [9, 10]. The method compares the current with the desired situation to formulate improvement measures and realise sustainable change.

While the intake in the morning by the department of OMS and the department of ENT remained the same, the MFDC in the afternoon of the same day served as an extra effort among the two front office departments OMS and ENT and the department Radiotherapy. The four contributing specialities are ENT, Radiotherapy, OMS and the Special Dental Care. Special Dental Care is a sub-department within the Department OMS. The MFDC aims to provide a preliminary diagnostic plan, with multidisciplinary agreement, stating the diagnostic procedures to be performed, so intake for treatment modalities could start as soon as possible. The patient is informed on his or her diagnostic plan at the end of the day.

We applied a mixed method study [11, 12] combining statistical results and interviews. Firstly in search for process indicators for care pathway management we evaluated throughput times and number of hospital visits, secondly we performed semi-structured interviews of involved specialists of core departments to explore outliers in throughput times until data-saturation was reached.

### Patients

The MFDC started in August 2007.

Four data sets were extracted, one data set of consecutive patients who were referred at least four months before the start of the MFDC (from April 2007 backwards), one data set of consecutive patients referred four months after the implementation of the MFDC (from January 2008 onwards) to compare immediate effects of MFDC. Two more datasets were extracted to analyse sustainability of the improvement over the five years after the implementation of the MFDC, one set of consecutive patients from January 2010 onwards and one set from January 2013 onwards.

Data of patients were included if they were 18 years of age and older, who had been curatively treated for a primary carcinoma of lips, oral cavity, oropharynx, nasopharynx, hypopharynx, or larynx (ICD(O) coding C00 through C14, C30 through C32) [13]. Data were excluded if patients were treated for an unknown primary tumour (C80), a second primary tumour in the head or neck region or if a recurrent or residual tumour was diagnosed.

### Process indicators and study design

The Dutch Cooperative Head & Neck Group developed the standard of ‘80% of the patients with a head and neck tumour start their primary treatment within 30 calendar-days from day of intake’, together with maximum throughput time for access to consultation and start treatment [2, 4]. For the evaluation of the effects of the implementation of the MFDC, the process indicators throughput times and number of hospital visits were used [1, 14–16]. We distinguished three different throughput times: the time to gain access to the first oncology consultation (access first consultation); the time to finish the diagnostic procedures, including the treatment plan (diagnostic procedures); and the time to start the first treatment (start first treatment). The throughput times ‘access first consultation’, ‘diagnostic procedures’ and ‘start first treatment’ were measured from the day the patients had their first oncology consultation in either one of the front offices of the centre. In the pre and post intervention situation in the centre, the consultation or intake was done once a week, independent of the number of patients referred (Fig. 1).

The first author registered the relevant data in a clinical registration form from electronic and written medical records. The last author checked the registrations of the medical records.

### Statistical analysis

Our primary outcome measure was the change in the throughput time to start the first treatment as a result of the intervention of implementing the MFDC. Initially the sample size was set at about 20 patient records in each period (2007 and 2008) as a starting point to evaluate management of the care pathway over the years. Based on an analysis of these samples we would determine the definitive sample size. However in the analysis significant differences were found in throughput times hence data acquisition regarding 2007 and 2008 was not continued. Additionally to analyse sustainability of data, records of 24 patients from 2010 and 24 from 2013 were used. Statistical analysis was performed using SPSS 23.0 for Windows software.

Analysis of variance was applied to outcome variables throughput time (referral, diagnostic procedures, start first treatment) and number of hospital visits (total, from intake to diagnostic procedures complete, from diagnostic procedures complete to start treatment), ‘age at start treatment’. Because samples were small and assumptions were not met, biased corrected bootstrapping (2000 samples) was applied [17]. The exact chi-square test was used to analyse differences in descriptive variables between the groups, regarding gender, tumour localization and tumour size, and compliance to the Dutch 30-day standard.

In all analyses, statistical significance was set at the 5% level.

### Qualitative analysis

Semi-structured interviews were used to explore reasons for non-compliance to the Dutch 30-day standard for starting the first treatment. Therefore the cutting point for ‘outliers’ chosen was defined as ‘longer than 37 days to start treatment’ (years 2008, 2010 and 2013); reflecting non-compliance to the Dutch 30-day standard and a (patient) delay of one week; for example if the first opportunity for outpatient clinic was skipped, either by the patient or for another reason.

We used the outlier cases to start the semi-structured interviews with one representative of each of the four departments that work together in the care pathway to give primary treatment (ENT, OMS, Radiotherapy and Medical Oncology). Prior to the interview the specialists were given the medical records of the outlier(s) to enhance recalling the case. Each semi-structured interview with a specialist started after getting verbal informed consent of the interviewees by providing them with information about the outliers. The interviewer (first author) then guided the interview using a short topic list including ‘cause of the delay’ and ‘perceived possibilities for change or improvement in logistics or of the care pathway’. The topics were introduced in a flexible way, and the interviews took the form of natural conversations.

The interviews were audio recorded and field notes were taken. Verbal transcripts of the interviews were made with the transcription program F4. The interviews lasted from 25 to 40 min. Quotations, related to throughput time or number of hospital visits, or improvement of the care pathway, were numbered in chronology of the interview. The first stage of the inductive analysis of interviews involved the last author and third author, in an open, initial coding procedure that resulted in a list of codes corresponding closely to the text fragments extracted from the four interviews. The codes were placed in a coding tree with major and minor themes in relation to management of the care pathway (Table 4) [18]. Any disagreements about the codes were discussed between the coders and the first author [19].

## Results

### Quantitative analysis

In total 89 medical records were included in the study: 21 in the ‘pre MFDC group, year 2007’ and 68 in the ‘post MFDC group, year 2008-2010-2013’ (Table 2). Two-thirds of the groups were men, with a mean age of 66 years. The tumours were located in the oral cavity (tongue, gums or floor of mouth), the salivary glands, oropharynx, nasopharynx, hypopharynx, or larynx. The primary tumour classification ranged from T1 to T4 [20]. We found no significant differences between the pre MFDC group and the post MFDC group in patient and tumour characteristics.

**Table 2:** Patient and tumour characteristics

	*Pre-MFDC*		*Post-MFDC*						Sign.
	*2007 (n = 21)*		*2008 (n = 20)*		*2010 (n = 24)*		*2013 (n = 24)*		ANOVA
**Age** Mean(SD)	66	(11)	66	(13)	63	(13)	64	(9)	.640
									Chi^2^
**Gender**	*n*	*%*	*n*	*%*	*n*	*%*	*n*	*%*	.680
Male	14	67	13	65	18	75	14	58	
**Tumour localization**									.303
Lip	0	0	0	0	0	0	2	100	
Oral cavity	8	38	11	55	17	71	9	38	
Tongue (C01, C02)	3		2		6		1		
Gums (C03)	1		3		2		0		
Floor of mouth (C04)	3		4		4		6		
Oral cavity, unspec.	1		2		5		2		
(C00, C05, C06, C14)									
Major salivary glands (C07, C08)	1	5	0	0	0	0	0	0	
Oropharynx (C09,C10)	2	10	2	10	1	4	4	17	
Nasopharynx (C11)	1	5	2	10	1	4	1	4	
Nasal Cavity (C30)	0		0	0	1	4	1	4	
Hypopharynx (C12, C13)	3	14	1	5	0	0	5	21	
Larynx (C32)	6	29	4	20	4	17	2	8	
**Tumour size**									.522
T1	9	43	8	40	10	42	4	17	
T2	5	24	4	20	5	21	6	26	
T3	3	14	2	10	3	13	2	8	
T4	4	19	6	30	5	21	12	50	
Tx	0	0	0	0	1	0	0	0	

In bold main patient characteristics of the dataset (age, gender, tumour localization and size)

**Fig. 2:**
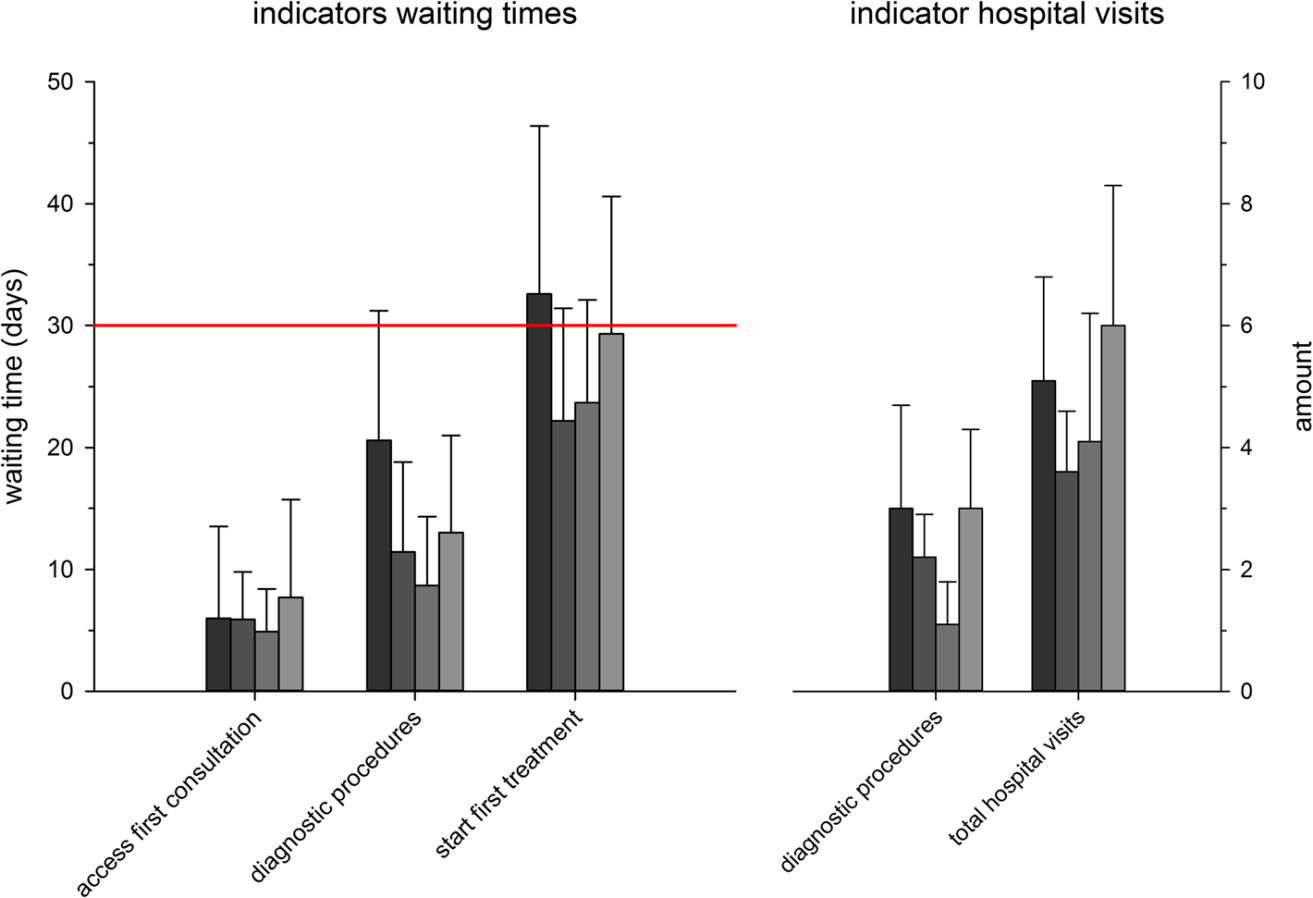
Throughput times and hospital visits pre- and post MFDC. Legend Fig. 2. Red line = the Dutch 30-day standard. Darkest grey bar = pre MFDC situation, year 2007. Dark grey bar = post MFDC situation, year 2008. Lighter grey bar = post MFDC situation, year 2010. Lightest grey bar = post MFDC situation, year 2013. Hospital visits is shown as hospitals visits from intake to completion of ‘diagnostic procedures’ and as ‘total hospital visits’

Throughput times for the diagnostic procedures and start treatment decreased significantly, with an average of eight days, after the implementation of the MFDC (comparison between 2007 and 2008) through the extra effort of the four specialist disciplines while no increase in personnel capacity was possible in the care pathway. Time to gain access to the first oncology consultation did not change significantly (Fig. 2 and Table 3).

**Table 3:** Throughput times and hospital visits, pre- and post-MFDC

	Pre	Post			Significance	
	2007 (*n* = 21)	2008 (*n* = 20)	2010 (*n* = 24)	2013 (*n* = 24)	*p* ANOVA	pair wise comparison
Throughput time (days)						
	Mean (SD)	Mean (SD)	Mean (SD)	Mean (SD)		
Access first consultation	6.0 (7.5*)	5.9 (3.9)	4.9 (3.5)	7.7 (8.0)	.592	–
Diagnostic procedures	20.6 (10.6)	11.4 (7.4)	8.7 (5.6)	13.0 (8.0)	<.000	2007–2008: .013; 2007–2010: .000; 2007–2013: .049
Start first treatment	32.6 (13.8)	22.2 (9.2*)	23.7 (8.4)	29.3 (11.3)	.009	2007–2008: .038
Number hospital visits						
Diagnostic procedures	3.0 (1.7)	2.2 (0.7*)	1.7 (0.7)	3.0 (1.3)	<.000	2007–2010: .014; 2008–2013: .049; 2010–2013: .001
Diagnosis to start Treatment	2.1 (1.5)	1.4 (0.9*)	2.4 (1.9)	3.1 (2.2)	.032	2008–2013: .012
Total	5.1 (1.7)	3.6 (1.0*)	4.1 (2.1)	6.0 (2.3)	<.000	2007–2008: .006; 2008–2013: .000; 2010–2013: .021
Start treatment within 30 days	52%	83%	71%	54%	.132	2007–2008: .040

Legend Table 3. Access first consultation; throughput time from ‘date of the letter of referral’ to ‘intake in oncology front office’, diagnostic procedures; throughput time from ‘first consultation’ to ‘decision in multidisciplinary meeting of the head & neck cancer centre’, start first treatment; ‘throughput time form first consultation’ to ‘start first primary treatment’. Hospital visits are measured during the diagnostic procedures, and from diagnosis to start treatment, and in total

*= number of patients is 19, because one patient was treated elsewhere after receiving the diagnostic plan

The number of hospital visits during the diagnostic phase reduced significantly with one visit (*p* < .032) after the implementation of the MFDC (Table 3). Furthermore we analysed hospital visits during the diagnostic phase and from diagnosis to start treatment; for 2013 we saw an increase for hospital visits during the diagnostic procedures and an increase in the total number of hospital visits (Table 3).

Before implementation of the multidisciplinary first-day consultation, treatment started for 52% of new patients within 30 calendar-days after the first consultation. After implementation of the multidisciplinary first-day consultation, this percentage increased significantly to 83% (*p* < .040). This percentage decreased again in 2010 and 2013. In 2013 waiting time to start treatment increased for all treating modalities, therefore outliers were analysed. In total, we defined 8 cases (12%) as ‘outlier’.

### Qualitative analysis – Specialist interviews

The specialists spontaneously gave their view on the agreements in the care pathway and described changes in guidelines, such as new treating modalities that may have increased throughput time.

In some cases the different aspects of a quotation was scored separately (Table 4). The interviews gave in total 76 scores, 37 quotations that were coded with 19 codes. Quotations appear in the text in italic.

**Table 4:** Codes in coding tree in relation to the care pathway management

Coding tree			Code	Code description	Frequency
Care pathway	Intake	Referral	1	Suspicion ‘malignity’ at intake not sufficient	9
		Patient related	2	Waiting on family home caregiver	3
			3	Co-morbid or complex patient	9
	Diagnostic procedures and logistics	Throughput time	6	More attention to cooperation between disciplines to combine patient appointments	6
			13	Control/logistics control lies with gate specialist or ‘core specialist’	6
			8	Treatment of dental foci under anaesthesia	2
		Waiting time	17	Waiting time Radiology	3
			18	Waiting time Nuclear Medicine	2
	Treatment and planning	Preparation	4	For pre-surgery assessment the treatment must be known, that is possible when staging of tumour is ready	3
		Choice	14	Choice for treatment on basis of general health assessment	1
			12	Scientific Research increases number of hospital visits	2
		Planning	5	Planning reconstruction costs extra time	5
			7	Planning capacity operation centre versus ‘examination under anaesthesia’- scopy	5
			11	Reconciliation of patient on chemo-radiotherapy	8
		Standardizing	16	Unclear starting moment waiting time chemo-radiotherapy, separate standard ‘Nederlandse Vereniging voor Radiotherapie en Oncologie’ (Dutch Association Radiotherapy and Oncology)	3
	Case Management Diagnostic procedures and treatment	Transfer	10	Transfer of ‘core specialism’	1
		Information	9	No management information on throughput times in electronic patient dossier	3
			15	No standard patient tracking system	4
		Registration	19	Registration information not clear	1
Total quotations 37					76

This coding tree has major and minor themes that were derived from the primary research question (intervention in management of the care pathway) and minor themes derived during coding

Analysing the interviews we learned that:

- introduction of new cure modalities chemotherapy and chemo-radiation took more preparation time and more hospital visits, coded as: ‘Planning reconstruction costs extra time’.

*‘Duration and severity of surgery is not only the dissection of the tumour, but also the reconstruction that is discussed in the reconstruction meeting, like an obturator or a flap.’*

*‘The treatment date is known [date], but clearly there were not enough slots in the ‘major surgery planning’ to treat this patient in time.’*

- specialists were not aware that throughput times were lengthening, because information on throughput times is not easily available in the electronic patient dossier, coded as: ‘No management information on throughput times in electronic patient dossier’.

*‘The gate specialist department agreed that they were supposed to keep track of throughput times, although this agreement was not traceable in writing.’*

*‘Register more accurately the throughput time when time to start treatment is longer than the 30-day standard.’*

*‘BROC (database for oncology registration) is only meant for basic tumour registration, not for management information on quality indicators.’*

One of the specialist departments tried to reduce throughput times by creating time slots at the imaging departments, coded as: ‘Waiting time Radiology or Nuclear Medicine (imaging)’.

*‘The slots are for radiotherapy patients at the Nuclear Medicine and Radiology department, for which hopefully in the future more slots become available in order to get PET-CT planned earlier. This is a logistic matter, which means that the amount of patients that need imaging to fit in the available slots is variable, sometimes only 2 and sometimes up to 10 patients. Back then we had less slots available.’*

Throughput times reduced again, but because slots at the imaging department were on consecutive days, rather than the same day, the number of hospital visits increased.

- co-morbid patients or patient delay took more time than expected (in 2013 62% of all patients started their treatment within the Dutch 30-day standard), coded as ‘Co-morbid or complex patient’.

*‘Madam is admitted to a nursing home and has a long history – co-morbidity.’*

- the interviewees saw opportunities for improvement of the care pathway, coded as: ‘More attention to cooperation between disciplines to combine patient appointments’.

*‘Nowadays we do not wait for PEG-placement to start treatment. During admission for the first chemotherapy, a PEG-tube can be placed.’*

### Combining quantitative and qualitative results

Effects of the implementation of the MFDC diminished in 2013 mainly because of the use of newer treatment modalities such as primary radiotherapy and chemo-therapy (from 32.6 days in 2007 to 22.2 days in 2008, and 23.7 days in 2010 back to 29.3 days 2013 on average, Table 3). In some cases patients needed extra time due to personal circumstances, in other cases preparation of a more complex treatment took more time and more hospital visits than in 2008 and 2010.

Each specialist gave his or her view on improving management of the care pathway when asked, they mentioned: *‘planning that cannot be influenced, when slots are not available’, ‘access of management information’* and *‘definitions of medical registrations’*.

## Discussion

We found that throughput times for diagnostic procedures and start treatment decreased considerably, with about eight days during the first years after MFDC implementation in 2007. The reduction in throughput times was a result of better logistics due to a multidisciplinary diagnostic plan, made during MFDC. There was no effect on referral times, because the MFDC is organized once a week. In 2008 the care pathway was in compliance with the Dutch national standard of 80% of new patients starting their treatment within 30 calendar-days after oncology intake. The patients visited the hospital approximately one time less, during the diagnostic phase. As a consequence of the introduction of the MFDC, the extra efforts of four specialist departments, a special care dentist, a nurse practitioner, and a coordinating nurse seeing the patient together during intake, were justified.

However when analysing sustainability through 2010 and 2013 we found that throughput times for diagnostic procedures were sustainable, but not for start treatment. Besides that the number of hospital visits for diagnostic procedures and hospital visits in total increased in 2013 (Table 3). From the outlier-evaluation we learned that there were four major themes in the coding tree: intake, diagnostic procedures and logistics, treatment and planning, and case management for diagnostic procedures and treatment. Complex treatment and co-morbid patients at intake took more time. Logistics and planning during the diagnostic phase were more difficult with complex treatment, more diagnostic or imaging needed to be planned and treatment with cooperation of different specialist departments were difficult to plan on the same day. Dental foci treatment can only be performed when the total treatment plan is finished, but slows down the process of planning for start treatment. New features as 3D-planning for surgery give better results [21], but increase time to start treatment. For patients that need the most complex procedures planned, case management for that individual patient, tracking and tracing for all disciplines, would be helpful to keep the throughput time at a minimum. In most of these cases management information was not available and the involved specialists were not aware that the throughput times increased. This shows the added value of the MFDC in reducing the time needed for the diagnostic procedures for complex care.

In support of the above Ouwens et al. demonstrated in 2007 [22] and 2009 [23] that integrated care for head and neck cancer patients results in an improvement of perceived quality of care by improving patient centeredness in organizational issues like reducing waiting times and medical-technical quality of the diagnostic equipment. According to the guideline, patients need a treatment plan delivered by a multidisciplinary team of a cancer centre and an evaluation of the execution of that treatment plan registered in the patient dossier. To follow those guidelines for head and neck cancer it is of utmost importance for management of cancer centres to have throughput time and amount of hospital visit information available at all times [20].

Coordination of the logistics diagnostic procedures is important to shorten the time until the start of treatment. Time slots for diagnostic procedures can help improve efficiency of the care pathway and start treatment earlier [10, 24, 25].

We think that in our study we have shown that the MFDC for head and neck patients is an added value: implementation improved efficiency (reduced throughput times and hospital visits) and compliance to the Dutch 30-day standard. Therefore, when management of logistics of the care pathway can be trusted for 80% of patients, specialists can use the multidisciplinary patient meetings to have collegial discussions on complex cases and keep focus on patient centeredness.

We decided to include patients in a certain time period around the intervention to reduce bias. The moment it was decided that the MFDC would start on a certain date, changes may occur in procedures and patient selection. After the implementation of the MFDC it is likely that there is a learning curve. Therefore we chose an eight-months period, four months before and four months after the implementation of the MFDC, in which no data was gathered.

To evaluate the MFDC implementation we chose throughput times as process indicators because they are often regarded as logistic management measures and used as a ‘benchmark’ for several purposes. Governmental bodies around the world try to compare their quality of oncological care with indicators such as necessary infrastructure and volume, and throughput time with other countries [20, 26–30]. However, direct relation between throughput times and outcome for head and neck cancer patients in our hospital is not proven. We chose to follow the Dutch standards that advice to use registrations on new patients with certain malignity only, that we called ‘with curative intent’. Our indicators for process efficiency (throughput times) were chosen in a framework for measuring quality by assessing elements of structures or processes with proven connections with key outcomes of interest [31–34]. A good quality or process indicator signals changes in quality and is registered in a reliable manner [14, 30]. Implementation of structural planning of diagnostic procedures for head and neck cancer patients was found to have a positive effect on throughput times: time slots as a logistic structure reduced the diagnostic phase for head and neck cancer patients [25]. The structural planning of slots for diagnostic procedures are appreciated by patient associations and are reflected in their description of process indicators [35]. Several studies have shown that clinical characteristics of patients and prognostic factors explain a relatively large part of the variation in outcomes, such as survival and quality of life, while quality-of-care indicators explain a much smaller part [36–38]. Monitoring the process of care in a clinical pathway in direct relation to assessment of quality of care is of major importance to benchmark complex care such as head and neck cancer, but is difficult to assess [2, 39–41].

We wanted to show with a small sample and a simple method to evaluate, the effect of an intervention in the care pathway on efficiency. The added value of the extra multidisciplinary patient meeting is proven. We think that the combination of process indicators throughput time and number of hospital visits can be used in a dashboard to help care pathway management to monitor and sustain the agreements made.

The results of this study show that a ‘simple’ intervention, such as the implementation of the MFDC, can improve throughput times directly, which in turn can help improve the perceived quality of care. Especially with complex, life-threatening diseases such as head and neck cancer, shortening of the pathways diagnostic procedure is important so that treatment can start as early as possible to give patients a better chance of survival [3, 23, 42, 43].

In case of low-volume, highly complex care such as head and neck cancer, patients are treated in a centre with large adherence area, about 11,400 km^2^ with a total of 2.3 million inhabitants for our centre. Because of travel distances, reducing hospital visits with one visit is a valuable contributor to patient comfort and cost reduction. A decrease of time of uncertainty about diagnosis, treatment and prognosis also reduces patient anxiety and increase patient satisfaction [35].

The reduction in throughput time was achieved mainly in the diagnostic phase of the care pathway. Although this study did not aim to improve a specific phase before start treatment, the time between the end of the diagnostic phase with the treatment plan and the start of the treatment has become relatively long. We recommend examining production agreements or slots for planning with medical support departments to further reduce the time to start treatment, thus reducing the risk of upstaging even more.

The reduction in throughput times was a result of better logistics due to a multidisciplinary diagnostic plan, made during MFDC. Management of the care pathway can use these indicators to stay focused on sustainable, seamless processes of care in a multidisciplinary setting [40, 44]. We would like the information needed for care pathway management to become available through our electronic patient dossier and in a dashboard, so that lengthening of throughput times could be detected before they become unacceptably high. In case of change in the described process indicator combination from agreed levels, the management should look for variation or deviation on the agreements on the care pathway (with best intentions made) that could influence future patient outcomes.

## Conclusions

We showed that the MFDC implementation in the care pathway had a positive effect on efficiency in the care pathway. As a consequence, the extra efforts of four specialist departments, a special care dentist, a nurse practitioner, and a coordinating nurse seeing the patient together during intake, were justified. Start treatment times increased as a result of new treatment modalities that needed more time for preparation.

## Abbreviations

BROC: Basic Registration Oncology outCome (database)
ENT: Ear, Nose & Throat
F4: Transcription program
ICD(O): International Classification of Diseases (of Oncology)
MeSH: Medical Subject Headings
MFDC: Multidisciplinary first-day consultation
MO: Medical Oncology
MPM: Multidisciplinary Patient Meeting
NWHHT: Nederlandse Werkgroep Hoofdhalstumoren or Dutch Cooperative Head & Neck Group
OMS: Oral and Maxillofacial Surgery
PEG: Percutaneous Endoscopic Gastronomy
PET-CT: Positron Emission Tomography – Computer Tomography
RT: Radiotherapy
SPSS: Statistical Package for Social Sciences
UMCG: University Medical Center Groningen

## References

[1] Ouwens M, Hermens R, Hulscher M, Vonk-Okhuijsen S, Tjan-Heijnen V, Termeer R, Marres H, Wollersheim H, Grol R (2010). Development of indicators for patient-centred cancer care. Support Care Cancer.

[2] Dutch Cooperative Head & Neck Group. Policy head and neck cancer care 2013 (in Dutch). 2013. http://www.nwhht.nl.

[3] Wyatt RM, Beddoe AH, Dale RG (2003). The effects of delays in radiotherapy treatment on tumour control. Phys Med Biol.

[4] Lingsma HF, Dippel DW, Hoeks SE, Steyerberg EW, Franke CL, van Oostenbrugge RJ, de Jong G, Simoons ML, Scholte Op Reimer WJ (2008). Netherlands stroke survey investigators. Variation between hospitals in patient outcome after stroke is only partly explained by differences in quality of care: results from the Netherlands stroke survey. J Neurol Neurosurg Psychiatry.

[5] Dutch Cooperative Head & Neck Group (2004). Evaluation report on bottle necks in head and neck cancer care (in Dutch).

[6] Dutch Cancer Registration. Cijfers over kanker / Figures on cancer. In: Nederlandse Kankerregistratie; 2016. https://www.cijfersoverkanker.nl. Accessed 6 May 2017.

[7] Kruijff S (2017). Het MDO toe aan een flinke renovatie. / The Multidisciplinary Meeting ready for a substantial renovation.

[8] Brunner M, Gore SM, Read RL, Alexander A, Mehta A, Elliot M, Milross C, Boyer M, Clark JR (2015). Head and neck multidisciplinary team meetings: effect on patient management. Head Neck.

[9] Lodewijckx C, Decramer M, Sermeus W, Panella M, Deneckere S, Vanhaecht K (2012). Eight-step method to build the clinical content of an evidence-based care pathway: the case for COPD exacerbation. Trials.

[10] van Gemert AG, Ahaus CTB, de Kleine AW, Franke HS, Kaagman CBM, CHE K (2012). Factors stimulating chain development in emergency care (Dutch: Bevorderende factoren voor ketenontwikkeling in de spoedzorg). KiZ.

[11] Plochg T, Juttman RE, Klazinga NS, Mackenbach JP (2007). Handbook health research (Handboek gezondheidszorgonderzoek): 1st ed.

[12] Charmaz K (2006). Contructing grounded theory. A practical guide through qualitative analysis.

[13] Vlaams Kankerregistratienetwerk. International classification of diseases for oncology, third edition, updates (in Dutch): Vlaams Kankerregistratienetwerk; 2011. whofic@who.int; ICDO3@iarc.fr ed

[14] Wollersheim H, Hermens R, Hulscher M, Braspenning J, Ouwens M, Schouten J, Marres H, Dijkstra R, Grol R (2007). Clinical indicators: development and applications. Neth J Med.

[15] Jaspers GW, Witjes MJ, Groen HJ, Groen H, Rodiger LA, Roodenburg JL (2011). Strategies for patients with newly diagnosed oral squamous cell carcinoma and a positive chest CT. A cohort study on the effects on treatment planning and incidence. Eur J Surg Oncol.

[16] Sobin LH, Gospodarowicz MK, Wittekind CE (2009). TNM Classification of Malignant Tumors, 7th ed.

[17] Wright DB, London K, Field AP (2011). Using bootstrap estimation and the plu-in principle for clinical psychology data. JEP.

[18] Tong A, Sainsbury P, Craig J (2007). Consolidated criteria for reporting qualitative research (COREQ): a 32-item checklist for interviews and focus groups. Int J Qual Health Care.

[19] Gioia DA, Gorley KG, Hamilton AL (2012). Seeking quality rigor in inductive research: notes on the Gioia methodology. Organ Res Methods.

[20] Dutch National Cancer Control Programme (2010). Progress Report on Cancer Control in the Netherlands, 2005–2010 (Dutch NCCP, Nationaal programma kankerbestrijding).

[21] Schepers RH, Kraeima J, Vissink A, Lahoda LU, Roodenburg JL, Reintsema H, Raghoebar GM, Witjes MJ (2016). Accuracy of secondary maxillofacial reconstruction with prefabricated fibula grafts using 3D planning and guided reconstruction. J Craniomaxillofac Surg.

[22] Ouwens MM, Marres HA, Hermens RR, Hulscher MM, van den Hoogen FJ, Grol RP, Wollersheim HC (2007). Quality of integrated care for patients with head and neck cancer: development and measurement of clinical indicators. Head Neck..

[23] Ouwens MM, Hermens RR, Hulscher MM, Merkx MA, van den Hoogen FJ, Grol RP, Wollersheim HC, Marres HA (2009). Impact of an integrated care program for patients with head and neck cancer on the quality of care. Head Neck..

[24] de Bree R, Veenvliet P, van Driel M, Verdonck-de Leeuw I, Goverde K, Koense YJ, Bijvank E, Leemans CR (2007). Development of a clinical pathway for head and neck cancer patients (in Dutch). Nederlands tijdschrift voor oncologie.

[25] de Bree R (2007). Booking recommended (in Dutch). Medisch Contact.

[26] Olesen F, Hansen RP, Vedsted P (2009). Delay in diagnosis: the experience in Denmark. Br J Cancer.

[27] Berrino F, De Angelis R, Sant M, Rosso S, Bielska-Lasota M, Coebergh JW, Santaquilani M, EUROCARE Working group (2007). Survival for eight major cancers and all cancers combined for European adults diagnosed in 1995-99: results of the EUROCARE-4 study. Lancet Oncol.

[28] Malin JL, Schneider EC, Epstein AM, Adams J, Emanuel EJ, Kahn KL (2006). Results of the National Initiative for Cancer care quality: how can we improve the quality of cancer care in the United States?. J Clin Oncol.

[29] Chen F, Puig M, Yermilov I, Malin J, Schneider EC, Epstein AM, Kahn KL, Ganz PA, Gibbons MM (2011). Using breast cancer quality indicators in a vulnerable population. Cancer.

[30] Mainz J (2003). Developing evidence-based clinical indicators: a state of the art methods primer. Int J Qual Health Care.

[31] Donabedian A (1992). Quality assurance. Structure, process and outcome. Nurs Stand.

[32] Donabedian A (1992). The role of outcomes in quality assessment and assurance. QRB Qual Rev Bull.

[33] Donabedian A (1988). 20 Years of Research on the Quality of Medical Care, 1964-1984. Salud Publica Mex.

[34] Mainz J (2003). Defining and classifying clinical indicators for quality improvement. Int J Qual Health Care.

[35] Offerman MPJ, Pruyn JFA (2009). Good care for people with head and neck cancer: quality criteria with a patients view (in Dutch: Goede zorg voor mensen met kanker in het hoofd-halsgebied: kwaliteitscriteria gezien van uit de patient.).

[36] Mant J (2001). Process versus outcome indicators in the assessment of quality of health care. Int J Qual Health Care.

[37] Landon BE, Zaslavsky AM, Beaulieu ND, Shaul JA, Cleary PD (2001). Health plan characteristics and consumers’ assessments of quality. Health Aff (Millwood).

[38] Donabedian A (1990). Contributions of epidemiology to quality assessment and monitoring. Infect Control Hosp Epidemiol.

[39] De Bleser L, Depreitere R, De Waele K, Vanhaecht K, Vlayen J, Sermeus W (2006). Defining pathways. J Nurs Manag.

[40] Vanhaecht K, De Witte K, Panella M, Sermeus W (2009). Do pathways lead to better organized care processes?. J Eval Clin Pract.

[41] Panella M, Marchisio S, Di Stanislao F (2003). Reducing clinical variations with clinical pathways: do pathways work?. Int J Qual Health Care.

[42] Waaijer A, Terhaard CH, Dehnad H, Hordijk GJ, van Leeuwen MS, Raaymakers CP, Lagendijk JJ (2003). Waiting times for radiotherapy: consequences of volume increase for the TCP in oropharyngeal carcinoma. Radiother Oncol.

[43] Huang J, Barbera L, Brouwers M, Browman G, Mackillop WJ (2003). Does delay in starting treatment affect the outcomes of radiotherapy? A systematic review. J Clin Oncol.

[44] Bradley PJ (2012). Multidisciplinary clinical approach to the management of head and neck cancer. Eur Arch Otorhinolaryngol.

